# Governing the attention dividend: AI-reclaimed clinician capacity as a health policy resource

**DOI:** 10.1093/haschl/qxag197

**Published:** 2026-08-01

**Authors:** Yusuke Shono

**Affiliations:** Tamatani Clinic, Osaka 533-0004, Japan

**Keywords:** ambient artificial intelligence, attention dividend, AI governance, primary care, clinical documentation, clinician capacity, health policy, care delivery, documentation burden, continuity of care, health equity, quality measurement, health system governance, safety-netting, payment models

## Abstract

Ambient artificial intelligence (AI) is reducing documentation burden in primary care, with real but modest and variable effects: reclaimed minutes in some settings, reduced cognitive load in others, and no guarantee that either reaches patients. This article argues that the resulting capacity—the attention dividend—should be governed as a health policy resource. Without deliberate allocation, it defaults to throughput, administrative absorption, and already-advantaged patients. The article specifies the payment and care model conditions that make deliberate reallocation more feasible, including hybrid and value-based payment, continuity add-on payments, monthly per-patient care-management payments, and primary care spending floors; proposes 3 priority uses—recognition of overlooked patients, continuity, and safety-netting and reassurance; and pairs 8 allocation questions with measurable indicators, concrete policy and operational mechanisms, and accountable actors across health system leaders, payers, purchasers, primary care practices, AI vendors, regulators, and accreditors. Seven named capture mechanisms describe how the dividend fails to reach patients, each with a corrective governance response. The policy question is not whether ambient AI saves time, but whether health systems govern the capacity it returns—through mechanisms that can be named, measured, and assigned.

Key Takeaways

- AI-reclaimed clinician capacity—time, reduced cognitive load, or both—is a governable health policy resource; left ungoverned, it defaults to throughput, administrative absorption, and already-advantaged patients.
- Reallocation begins with payment: hybrid and value-based models, continuity add-on payments, monthly per-patient care-management payments, and primary care spending floors reduce the default pull toward visit volume.
- Every allocation question should be paired with a measurable indicator, a concrete mechanism, and an accountable actor—protecting recognition, continuity, and safety-netting and reassurance, with equity-stratified reporting and named capture mechanisms audited over time.

## Introduction

Artificial intelligence (AI) is entering primary care with a familiar and compelling promise: less documentation, less administrative burden, and more time for patients. If AI can reliably reduce clerical friction and lighten the cognitive work of documentation, that help should be welcomed rather than resisted. The relevant policy question, however, is not whether AI can save documentation time. It is what happens, at the level of health systems, to whatever capacity AI creates.

The evidence to date shows effects that are real but modest and variable. In a longitudinal cohort study across 5 US academic health systems, adoption of ambient AI scribes was associated with a 13.4-minute reduction in total electronic health record time and a 16.0-minute reduction in documentation time per 8 scheduled patient hours, with the largest effects in primary care—and no statistically significant change in work performed outside scheduled hours.^1^ A multisystem evaluation found that 30 days of ambient AI scribe use was associated with a fall in clinician burnout from 51.9% to 38.8%, alongside improvements in cognitive task load, after-hours documentation, and undivided attention to patients.^2^ Two implications follow. First, ambient AI creates potential capacity—time, reduced cognitive load, or both—rather than guaranteed free minutes, and the size and form of that capacity vary by setting. Second, the most consistent gains may be in attention and cognitive load, even where clock time changes little. Either way, the question moves downstream: where does the reclaimed capacity go?

It rarely remains unclaimed. Minutes that appear to be “freed” can be reallocated, redirected, or recaptured by throughput targets, inbox messages, reporting demands, coding requirements, and rising productivity expectations.^3-5^ The technology itself is only the mechanism that produces this capacity. What it creates is a new, largely invisible resource—reclaimed clinician capacity—whose destination remains largely ungoverned.

This is not an argument against access gains. In a primary care landscape strained by workforce shortages, rising panel complexity, and unmet need, the prospect that AI may expand visit capacity or shorten waits is a genuine benefit. The question is not whether throughput should benefit from AI-enabled efficiency, but whether it should become the only destination—and whether that choice is made deliberately or by default.

In this article, the attention dividend refers to that surplus of usable clinician capacity—reclaimed time, reduced cognitive load, or both—treated as a health policy resource whose allocation is a policy choice rather than a technical default. Recent commentary has observed that ambient AI evaluation measures inputs and immediate outputs well while remaining limited in measuring whether those gains reach outcomes that matter for patients.^6^ This article supplies the governance mechanism that decides whether they do—naming which actor, through which lever, against which indicator, allocates the capacity AI returns.

## Reclaimed capacity as a policy resource

The attention dividend is not merely a metaphor. It refers to a potentially measurable gain in clinician capacity—produced when AI reduces documentation and adjacent administrative work—and, at scale across primary care, it carries the structural features of a health policy resource. Its size and form are shaped by the conditions of production: which tools are deployed, how implementation interacts with existing workflows, and how much of the gain arrives as minutes vs reduced cognitive load. Its distribution is shaped by the incentives and structures that surround it—payment models, productivity expectations, workforce constraints, and equity priorities. And its value depends entirely on where it ends up.

Temporal and cognitive gains are not interchangeable. When the gain is temporal, systems can allocate schedulable capacity; when it is primarily cognitive, governance should protect visit length and monitor cognitive task load and patient-reported attention rather than convert the gain into additional volume.

Reclaimed capacity can follow 2 broad destination pathways (Table 1). It can be captured by existing system demand—through increased throughput, revenue-generating activity, administrative absorption (including verification of AI-generated outputs), and rising baseline expectations. Or it can be returned to the forms of care primary care is uniquely positioned to provide—recognition of patients most easily overlooked, longitudinal continuity, and safety-netting through which uncertainty is held without abandonment. Equity is a cross-cutting allocation lens that asks who benefits within either pathway, with particular attention to patients whose communication style, language, multimorbidity, or social complexity makes them easier to overlook in routine workflows. The equity lens applies across both pathways; the policy question is whether the distribution reflects deliberate choice or organizational default.

**Table 1.** Resource flow framework: 2 destination pathways and an equity allocation lens for AI-reclaimed clinician capacity in primary care.

Captured by existing system demand	Returned to primary care value	Equity lens across both pathways
Increased throughput	Recognition of patients most easily overlooked	Patients with limited English proficiency
Revenue-generating activity	Longitudinal continuity	Patients with multimorbidity or complex needs
Administrative absorption, including verification burden	Safety-netting and reassurance through accountable follow-through	Patients in higher-deprivation contexts
Rising baseline expectations	Care coordination and safety-netting	Patients otherwise easier to overlook in routine workflows

*Implementation pathway*: Ambient AI implementation → reduced documentation burden and clerical friction → reclaimed clinician capacity (the attention dividend), a new health policy resource requiring deliberate allocation across the pathways shown.

Equity is a cross-cutting lens rather than a third pathway; the policy question is whether distribution across the pathways reflects deliberate choice or organizational default.

Treating the attention dividend as an optimization problem—how to maximize reclaimed time per session—misframes the policy question. Optimizing for the production of reclaimed time is a workflow question; allocating reclaimed time across competing destinations is a policy question. Health systems, payers, purchasers, primary care practices, AI vendors, regulators, and accreditors each hold a lever that shapes where the attention dividend lands. Made visible, those levers become governable. Left invisible, they default to whichever destination has the strongest pull in the existing payment, productivity, and workforce environment.

## Why primary care capacity needs governance

The case for deliberately reinvesting reclaimed capacity rests on what primary care is for. Primary care does not consist only of well-defined episodes of decision-making, nor of the discrete information transactions that AI-enabled efficiency most readily accelerates. It consists, at least as much, of recognition that accumulates over time, of patterns that become clear only across repeated encounters, and of relationships through which clinical judgment can be distinguished from clinical reaction. The classical map of primary care—first contact, comprehensiveness, continuity, and coordination^7^—still describes the conditions under which generalist care delivers lower costs, fewer inequalities, and better population health.^8^

In this setting, time is not merely an operational input; it is a clinical instrument. The problem presented in one visit can be reinterpreted by what surfaces in the next; an unresolved concern can return under a different label and only then disclose its real shape. A substantial body of evidence supports continuity as a core feature of primary care associated with better patient outcomes and experience,^9^ and a recent nationwide population-based cohort study has shown that even clinic-level continuity—not only individual-clinician continuity—is associated with meaningful reductions in adverse outcomes.^10^ Continuity is what allows a deviation from this patient's baseline to be recognized as a deviation rather than read as a generic presentation.

Much early enthusiasm for clinical AI has focused on information processing—the retrieval of guidelines, the summarization of charts, and the drafting of notes. These contributions are real, and primary care benefits from them. But primary care depends on knowing which facts matter in this life, at this moment, in this relationship; the policy destinations of reclaimed capacity must therefore protect the interpretation of what happens between encounters, not only the efficient handling of discrete events.

Primary care capacity is already being reshaped by converging forces: ownership change, with private equity acquisition altering workforce and utilization patterns^11,12^; payment that privileges billable activity over relationship-based work; and persistent workforce shortage, with US primary care burnout among the highest of comparable high-income countries.^13^ Ambient AI joins this list as a distinctive driver: it creates marginal clinician capacity rather than reorganizing existing ownership, payment, or workforce arrangements—yet the destination of that capacity remains largely ungoverned.

One objection deserves a direct answer: in a system driven by fee-for-service revenue and panel-size pressure, can attention be governed at all? The premise is correct, but it does not eliminate the need for governance. Those pressures are precisely why allocation defaults to throughput—and they operate through a narrow definition of productivity: visits, relative value units (a common measure of billable clinical work), encounters per session. Meaningful primary care productivity is broader. It includes continuity maintained, follow-up completed, concerns recognized before they escalate, and uncertainty managed safely—outputs that attention produces and can be measured and rewarded (Section “Concrete governance mechanisms and indicators”). The policy question is not attention vs productivity, but which definition of productivity payment and measurement reward. The next section turns to those conditions.

## Payment and care model conditions

Reclaimed capacity does not enter a neutral environment. Where payment is predominantly fee-for-service, revenue tracks visit volume, and panel size and access pressure create a standing queue for any capacity that appears. Under these conditions, the default destination of reclaimed time is additional visits. This is not a failure of intent; it is what the payment model prices. Predominantly fee-for-service payment rewards discrete encounters more consistently than recognition, continuity, or follow-up delivered outside visits. Governing the attention dividend therefore begins with payment and care model conditions.

Prospective payment, including capitated and other population-based models, can change that default. Hybrid models that blend monthly per-patient payments with a reduced fee-for-service component—the direction recommended by the National Academies for US primary care^14^—make nonvisit work financially visible: panel review, asynchronous follow-up, coordination, and outreach to patients who have not returned. Value-based primary care contracts can go further, attaching payment to continuity and completed follow-up rather than to visit counts alone.

The durable levers are classes of mechanisms rather than named programs. Five classes are most relevant here. First, continuity add-on payments: the US Medicare visit-complexity add-on code (G2211) pays clinicians for serving as the continuing focal point of a patient's care, though its uptake—appearing on roughly a quarter of eligible Medicare visits by mid-2025—suggests that a payment signal alone may not reorganize practice.^15^ Second, monthly per-patient care-management payments conditioned on closed-loop follow-up, meaning follow-up that is completed, documented, and assigned to a responsible team member. Third, primary care spending targets or floors adopted by at least 6 US states, which protect the budget envelope within which reallocation happens.^16^ Fourth, purchaser contract clauses requiring health systems to report how AI-enabled capacity was allocated. Fifth, value-based contracts that include continuity, follow-up completion, and equity-stratified measures. Recent US experience reinforces the emphasis on classes over programs: In 2025, the Centers for Medicare & Medicaid Services (CMS) announced the early termination of 2 federal advanced primary care models, even as a bundled monthly care-management payment introduced in the same period carried the underlying mechanism forward.^17^ Similar allocation pressures arise in other high-volume primary care systems, although the available levers differ by country.

Payment reform alone, however, does not allocate attention; it removes the strongest opposing pull. To reach patients, payment conditions must be paired with the operational and accountability mechanisms described below—scheduling protections, panel-registry review, vendor reporting requirements, and equity-stratified follow-up reporting. These conditions should protect clinician discretion rather than script it. The purpose is not to dictate what happens in a reclaimed minute, but to make it economically rational to spend that minute on recognition, continuity, and safety-netting—and to let clinicians decide, patient by patient, where attention is needed.

## Three priority uses of reclaimed capacity

If payment and care model conditions create room for reclaimed capacity, the next question is where that capacity should go first. Three uses have the strongest claim in primary care: recognition, continuity, and safety-netting and reassurance (Table 2).

**Table 2.** Three priority destinations for reclaimed clinician capacity, with associated policy questions and protections.

Destination	Policy question	What it protects	Example use or protection of reclaimed capacity
Recognition	Are overlooked patients receiving more clinician attention?	The capacity of primary care to notice patterns that routine workflows miss	Proactive contact with patients who have repeatedly missed visits, have multimorbidity, or present with nonspecific concerns
Continuity	Is the same-clinician or same-team relationship preserved as throughput rises?	The longitudinal interpretive function unique to primary care	Scheduling structures and panel management that preserve relational continuity over time
Safety-netting and reassurance	Is uncertainty held within the system through accountable follow-through?	Diagnostic safety in primary care; safe management of diagnostic uncertainty	Safety-net planning, results follow-through, and proactive re-contact for unresolved concerns

### Recognition

Under throughput pressure, the patients most likely to be missed are the ones not asking: the patient who stopped coming, the abnormal result that was never followed up, the gradual decline that never crossed an urgent threshold. Recognition means deliberately directing freed capacity toward these patients rather than waiting for them to surface. The operational form is concrete: a regular panel-registry review that lists patients with overdue follow-up, unresolved abnormal results, or repeated missed appointments—paired with protected clinician time to act on that list. This is the use of the attention dividend that most directly reaches patients who would otherwise remain invisible to the schedule.

### Continuity

Continuity—care from the same clinician or team over time—has the strongest evidence base of the 3. A 2025 systematic review concluded that higher personal continuity between general practitioners and patients is associated with lower mortality, fewer hospital admissions, and fewer emergency department visits.^18^ The largest single study, a Norwegian national registry analysis of more than 4.5 million patients, found that longer relationships with the same general practitioner were associated with progressively lower odds of death: compared with a 1-year relationship, the odds fell steadily with relationship length, reaching an odds ratio of 0.75 after more than 15 years, with parallel patterns for acute hospital admission and out-of-hours use.^19^ Continuity is particularly relevant for patients with multimorbidity, whose care requires judgments to be integrated across conditions, clinicians, and time.^20^ The allocation point is that continuity is produced upstream by scheduling. Reclaimed capacity can be released as generic access—more slots with whoever is free—or protected as same-team slots for a clinician's own panel; only the second builds continuity. Continuity, in other words, is a scheduling and staffing decision before it is a clinical one—which is precisely what makes it governable.

### Safety-netting and reassurance

Reassurance is sometimes heard as kind words at the close of a visit. What this article means is more structured, and it corresponds to safety-netting: an established primary care practice for managing diagnostic uncertainty by informing patients and organizing follow-up.^21-23^ In operational terms, it includes communicating negative or inconclusive results rather than leaving silence; explaining what has been ruled out and what remains uncertain; telling patients which symptoms should prompt a return, and when; planning follow-up rather than leaving it to chance; assigning responsibility for that follow-up to a named clinician or team member; and keeping unresolved concerns visible until they are resolved. A simple example is a follow-up call after a negative test result: explaining what the result does and does not rule out, what would change the assessment, and who remains responsible for follow-up. Reassurance is the patient-facing outcome of this structure—the patient knows what was found, what was not, and who is watching. That structure is also what separates reassurance from false reassurance: the safety lies in the follow-up, not in the tone. These tasks are the first to disappear under time pressure because they are brief, live between visits, and are rarely counted as productivity; they are also among the most direct conversions of reclaimed minutes into diagnostic safety.

## Concrete governance mechanisms and indicators

Measurement alone does not produce governance. A framework that asks only whether AI saves time, and how much, leaves the central allocation question untouched. The framework proposed in Table 3 begins instead with the resource allocation question—what is being decided? —and pairs each question with a measurable indicator, a concrete policy or operational mechanism, and the actor most accountable for it. Rather than asking health systems to demonstrate that ambient AI works, it asks them to demonstrate where reclaimed capacity goes—and who, in each case, holds the lever that determines the destination. This is consistent with broader calls for safe, effective, and trustworthy AI governance,^24^ and with evidence that AI and predictive models are already widely used and variably evaluated across US hospitals.^25^

**Table 3.** Policy indicators, concrete mechanisms, and accountable actors for the attention dividend in primary care.

Resource allocation question	Example indicator	Concrete policy or operational mechanism	Accountable actor(s)
What clinician capacity does ambient AI return—time, reduced cognitive load, or both—and what verification burden offsets it?	Net documentation minutes saved per scheduled hour, minus verification time spent on AI-generated outputs, reported separately from changes in cognitive task load	Vendor implementation agreements requiring standardized reporting of time use, verification burden, and cognitive task load as a condition of deployment; routine review of these measures in practice operations	AI vendor; health system leadership
Across which destinations is reclaimed capacity distributed?	Change from baseline in scheduled nonvisit care blocks, completed follow-up contacts, protected continuity slots, added visit slots, and documentation or verification time	Purchaser and payer contract clauses requiring periodic reporting on allocation of AI-enabled capacity; care model redesign that pre-assigns a share of reclaimed capacity to nonvisit care blocks	Purchasers; payers; health system leadership
Are overlooked patients receiving reclaimed clinician attention?	Follow-up completion rates for patients with repeated missed visits, multimorbidity, or repeated contacts without resolution	Monthly panel-registry review listing patients with overdue follow-up, unresolved abnormal results, or repeated missed appointments, with protected clinician time to act on the list	Primary care practices; health system leadership
Is longitudinal continuity preserved as throughput rises?	Same-clinician or same-team continuity index across panels using ambient AI, compared with preimplementation baseline	Protected same-team continuity slots built into scheduling templates; continuity add-on payments that reward longitudinal responsibility	Health system leadership; primary care practices; payers
Is uncertainty held within the system through accountable follow-through?	Documented follow-up contact for unresolved results or referrals within a locally defined interval; safety-net plan completion; closure rates on outstanding results	Named responsibility for each pending result or referral; standing recontact protocols for unresolved concerns; monthly per-patient care management payments conditioned on closed-loop follow-up	Primary care practices; payers; accreditors
Do patients experience the allocation as renewed primary care attention?	Patient-reported experience of being heard, continuity, and the sense that someone is following up on unresolved concerns	Inclusion of a patient-reported item—“someone is following up on my unresolved concern”—in existing experience-of-care surveys	Payers; purchasers; health system leadership
Does allocation widen or narrow equity?	Rows 3 and 5 stratified by language, age, multimorbidity, and area-level deprivation	Equity-stratified reporting required as a condition of value-based or AI-supported delivery redesign contracts	Payers; purchasers; regulators; primary care practices
Is workforce burden, including for nonclinician staff, accounted for?	Workload measures across nursing, support, and administrative staff during implementation; staff-reported new oversight or correction tasks	Workforce impact assessment before and after deployment; staffing adjustments funded before throughput expansion	Health system leadership; regulators; accreditors

Each row links a policy-level allocation question to a measurable indicator, a concrete mechanism through which that allocation can be enacted, and the actor(s) whose decisions most directly determine the outcome. The indicators are illustrative starting points requiring contextual adaptation and empirical validation before deployment as formal evaluation metrics; multiple actors may share accountability for a single mechanism in practice. Allocation indicators should be paired with balancing measures—appointment wait time, unplanned acute care use, total cost per attributed patient, and workforce burden.

Table 3 organizes 8 resource allocation questions that ambient AI implementation in primary care should be expected to answer. Each row follows the same 4-column structure: the policy-level question being decided, an example indicator that makes the choice measurable, a concrete policy or operational mechanism through which the allocation can be enacted, and the actor most accountable for it. The 8 questions cover the full arc from generation to allocation—from how reclaimed capacity is measured at the workflow level, through how it is distributed across destinations and patient groups, to how patient-experienced outcomes show whether the allocation reached patients. The framework is structural, not exhaustive. These indicators are not the article's central contribution in themselves; they function as examples within a broader allocation architecture that links measurement to governance responsibility.

The indicators fall into 2 types. Some are direct—net documentation minutes, follow-up completion rates—and computable from existing electronic health record infrastructure; others are necessarily proxies for relational substance, such as the patient-reported sense that someone is following an unresolved concern. A framework that relies only on direct indicators will systematically underweight the destinations primary care most needs to protect. Two cautions follow. Indicators that ignore verification burden overstate the size of the dividend. And patient-experienced measures are central rather than confirmatory: whether allocation reached patients cannot be answered from inside the workflow alone.

No single actor controls allocation, and a useful framework names who holds each lever. Health system leadership shapes care models and scheduling architectures; payers and purchasers—including employers in the US context—structure incentives and accountability requirements; primary care practices make day-to-day allocation decisions through scheduling, panel management, and follow-up protocols; AI vendors set implementation parameters, including how verification burden is structured; regulators and accreditors define minimum standards. The purpose is not to assign blame, but to make explicit which actor holds which lever.

### Equity-stratified allocation as an accountability requirement

Equity enters this framework as an allocation question, not only a performance one. Whether ambient AI performs equally well across patient groups is already an active concern in evaluation and regulation^6^; the governance-level question is what happens to reclaimed capacity once it exists. If it is not deliberately allocated, it will tend to flow toward patients who are already well-engaged and articulate rather than toward those whose needs are harder to articulate, longer to address, or easier to overlook in routine workflows. The allocation of reclaimed capacity is itself an equity event. Recent experience with remote physiologic monitoring suggests that uptake patterns are not technologically predetermined: between 2018 and 2022, adjusted use rose fastest among Black and Hispanic Medicare patients^26^—supporting the narrower proposition that implementation, outreach, and allocation rules shape who benefits. The accountability requirement is concrete: equity-stratified reporting of follow-up completion by language, age, multimorbidity, and area-level deprivation, attached as a condition of value-based or AI-supported delivery redesign contracts, with panel-registry outreach directed to the strata falling behind (Table 3).

The indicators remain illustrative starting points rather than validated instruments, requiring contextual adaptation and empirical testing before deployment as formal evaluation metrics. A health system need not adopt the full framework at once; it can begin with a single measurable starting point—net documentation minutes saved per scheduled hour, net of verification time—and add allocation, equity, and patient-experience indicators as governance capacity grows.

Recent commentary in this journal has proposed risk-stratified oversight, procurement standards, and postdeployment monitoring for autonomous AI actions.^27^ This article addresses a complementary object of governance: the clinician capacity that ambient AI returns.

## Failure modes and safeguards

A policy framework is most useful when its failure modes are explicit and nameable. Table 4 names 7 capture mechanisms through which the attention dividend can fail to reach patients—throughput capture, administrative absorption, continuity erosion, inequity widening, burden shift, baseline drift, and setting-specific misallocation—and pairs each with a corrective governance response. Naming matters: each name attaches a specific allocation failure to a specific response and an accountable actor. None of these mechanisms is an argument against ambient AI in primary care; each is an argument for making visible what allocation choices health systems are actually making with the capacity AI returns.

**Table 4.** Capture mechanisms: how the attention dividend fails to reach patients, and corrective governance responses.

Capture mechanism	What happens	Corrective governance response
Throughput capture	Reclaimed time is absorbed entirely by additional visit volume; the dividend is visible in throughput metrics but never reaches recognition, continuity, or safety-netting	Preassign a defined share of reclaimed capacity to nonvisit care; payment that rewards continuity and completed follow-up rather than visit counts alone
Administrative absorption	Time flows back into inbox volume, message triage, note correction, and verification of AI-generated outputs—pronounced early in implementation and persistent if administrative demand grows with efficiency	Cap verification workload through vendor implementation parameters; track net rather than gross minutes saved; scheduled review of administrative task growth
Continuity erosion	Efficiency is used to fragment care across more clinicians and briefer encounters; throughput rises while longitudinal interpretation falls	Protected same-team slots in scheduling templates; continuity indicators tracked against pre-implementation baseline
Inequity widening	Reclaimed attention flows to patients already well-engaged, while patients harder to reach—by language, communication style, or social complexity—gain nothing; amplified where AI tools perform variably across communication patterns	Equity-stratified reporting of follow-up completion; panel-registry outreach targeted to under-served strata
Burden shift	Clinician time is purchased at the cost of new, invisible oversight and coordination work for nursing, support, and administrative staff	Workforce impact assessment across all staff groups; staffing adjustments funded alongside deployment
Baseline drift	Efficiency is silently absorbed by rising expectations—more patients per session, more documentation, faster inbox response—a productivity ratchet that erases the dividend without a visible decision	Fix explicit pre-implementation baselines for panel size and session load; require that raising them be an explicit decision, not drift
Setting-specific misallocation	Capacity deployed in a new care setting is absorbed by that setting's existing demands because its allocation purpose was never defined	Define setting-specific allocation priorities and destinations before or early in each new deployment

Naming each failure mode attaches a specific allocation failure to a specific governance response; the accountable actors correspond to those in Table 3.

Governance itself has costs, and a framework that ignored them would repeat the error it criticizes. Reporting requirements can consume the very capacity they are meant to protect; protected continuity slots can lengthen waits for patients without an established clinician; spending floors constrain budgets elsewhere in the system. Three design principles limit these effects: prefer indicators computable from existing records over new documentation; scale reporting obligations to practice size; and treat effects on cost, access, and quality in adjacent areas as monitored outcomes of allocation policy rather than afterthoughts. Allocation indicators should therefore be paired with balancing measures—appointment wait time, unplanned acute care use, total cost per attributed patient, and workforce burden—so that gains in one domain are not purchased through losses elsewhere. Commentary in this journal on AI-enabled care-model redesign similarly warns that improved coordination may nonetheless increase total service volume under fee-for-service, reinforcing the need for such balancing measures.^28^

A final safeguard concerns who actually controls allocation. Policy actors do not direct any given clinical minute; clinicians do, patient by patient, shaped by practice style and judgment. What health systems, payers, purchasers, and vendors control are the conditions under which those micro-decisions occur—schedules, panel sizes, staffing, measurement, and incentives. The framework's aim is therefore not to micromanage clinical judgment but to set defaults and accountability conditions under which reclaimed capacity is not automatically captured—and then to leave the decision about where attention is needed with the clinician who knows the patient.

## What reclaimed capacity should protect

Throughout this framework, a quieter question persists: what protected attention is governance meant to preserve? Health systems across contexts have words for this kind of care. One such term, the Japanese concept of **te-ate**—literally “placing the hand,” commonly associated with hands-on care—offers one moral vocabulary for the protected attention at stake here. Te-ate is not a competing alternative to ambient AI, nor a nostalgia for pre-digital practice. It points to attention that is embodied, accountable, and addressed to the person whose life gives a clinical problem its meaning. Policy indicators and governance levers make allocation visible. Te-ate names what visible allocation should be *for*. Without that moral grounding, governance frameworks risk becoming mechanisms for optimizing the wrong thing.

## Conclusion—a policy call

The success of ambient AI in primary care should not be measured only by faster notes or higher visit volume. It should be measured by whether the capacity AI returns is deliberately reinvested in what primary care is uniquely positioned to do—recognition, continuity, and safety-netting and reassurance. The framework proposed here makes that reinvestment governable, pairing each allocation question with an indicator, a concrete mechanism, and an accountable actor.

That framework asks something specific of each actor: health system leaders, to design care models and schedules that let reclaimed capacity flow toward primary care value rather than throughput alone; payers and purchasers, including employers, to reward continuity and completed follow-up through the payment conditions described above; primary care practices, to build recognition of overlooked patients into panel management rather than leave it to discretionary attention; AI vendors, to design implementation parameters that account for verification burden rather than hide it; and regulators and accreditors, to set minimum standards for acceptable allocation.

Ambient AI will continue to enter primary care. The governing question is not whether it saves clinician time, but whether health systems direct the capacity it returns—deliberately, accountably, and through mechanisms that can be named—toward what primary care is for.

## Supplementary Material

qxag197_Supplementary_Data
